# Neural Stem Cells in the Adult Brain: From Benchside to Clinic

**DOI:** 10.1155/2012/378356

**Published:** 2012-11-05

**Authors:** Oscar Gonzalez-Perez, Jose M. Garcia-Verdugo, Alfredo Quinones-Hinojosa, Sonia Luquin, Graciela Gudino-Cabrera, Rocio E. Gonzalez-Castaneda

**Affiliations:** ^1^Neuroscience Laboratory, Psychology School, University of Colima, Avenue Universidad 333, 28040 Colima, COL, Mexico; ^2^Department of Neuroscience, University of Guadalajara, Sierra Mojada No. 950, 44340 Guadalajara, JAL, Mexico; ^3^Laboratorio de Morfologia Celular, Centro de Investigacion Principe Felipe, CIBERNED, 46031 Valencia, Spain; ^4^Department of Comparative Neurobiology, Instituto Cavanilles, University of Valencia, 46071 Valencia, Spain; ^5^Department of Neurosurgery, The Johns Hopkins Bayview Medical Center, Baltimore, MD 21224, USA; ^6^Department of Cellular and Molecular Biology University of Guadalajara, 45110 Zapopan, JAL, Mexico

Increasing evidence indicates that neural stem cells (NSCs) play an important role in sustaining cellular homeostasis and brain tissue restoration. The study of all mechanisms that control and modulate the function of NSC is a crucial step for the design of therapies against chronic neurodegenerative processes. In this special issue of the journal, we had the pleasure to edit the topic entitled “*Neural Stem Cells in the Adult Brain: From Benchside to Clinic.*” This special compilation of paper was aimed to provide a global forum for publications of original peer-reviewed manuscripts that reported original research findings in the field of adult neural stem cell, including short communications, full-length research, and review articles. Below, we briefly discuss the papers you may find in this issue.

K. Nakaguchi et al., in their paper entitled “Growth factors released from gelatin hydrogel microspheres increase new neurons in the adult mouse brain”, suggest that new neurons born in the subventricular zone (SVZ) may be able to replace neurons lost in degenerative disease or injury and improve or repair neurological deficits. In this excellent paper, they tested whether delivering growth factors via gelatin hydrogel microspheres can support neurogenesis in the SVZ. Their findings indicated that hepatocyte growth factor-containing microspheres increased the number of new neurons migrating from the SVZ towards the injured striatum in a stroke model in mouse. Therefore, they propose that gelatin hydrogel microspheres may be a good delivery tool for the sustained release of growth factors to promote neural regeneration of damaged brain tissues. 

J. Tu and S. O. Ugoya discuss that traumatic brain injury (TBI) is one of the leading causes of major disability and death worldwide. Neural stem cells (NSCs) have recently been shown to contribute to the cellular remodeling and may represent a possible therapy for TBI. In their work the authors nicely summarized a critical assessment of recent data and developed a view comprising of six points to possible quality translation of NSCs in TBI. 

B. P. Carreira et al., in their paper entitled “Regulation of injury-induced neurogenesis by nitric oxide”, sustain that nitric oxide (NO), a pleiotropic signaling molecule in the central nervous system, is able to modulate neurogenesis, acting as a pro- or antineurogenic agent. In their interesting review, they discussed the relevance of the NO system for the treatment of neurodegenerative diseases or several pathological conditions that affect the brain.

J. Namiki et al. analyzed the phosphorylation of nestin, an intermediate filament protein commonly used as a neural stem/progenitor cell marker. Nestin is required for the survival and self-renewal of neural stem cells. In this study, the authors nicely reported CNS-specific phosphorylation sites in nestin that allow distinguishing vascular expression of nestin from other intermediate filament protein subtypes.

Hormonal signals from the pancreatic islets influence the energy homeostasis of the brain and *vice versa*. In an excellent review, M. Machida and coworkers explain the correlation between the insulin-mediated regulatory system of the CNS and the pancreatic endocrine system. Remarkably, adult neurogenesis from undifferentiated neural stem cells is greatly decreased in diabetic patients, and as a result their learning and memory functions decline. In their paper, the authors summarized latest research regarding this endocrinal and neurological relationship.

L. Calatrava-Ferreras et al. explained that cerebellar ataxias, a heterogeneous group of diseases characterized by motor incoordination, may be treated using cell transplantations. Specifically they propose the use of human umbilical cord blood mononuclear cells as a promising approach for restoration of cerebellar function 

S. Martinez-Herrero et al. discuss that adrenomedullin (AM) acts as a growth and cell fate regulatory factor for adult neural stem cells. AM regulates the proliferation rate and the differentiation of stem/progenitor cells into neurons, astrocytes, and oligodendrocytes, probably through the PI3K/Akt pathway. AM gene is also able to regulate the cytoskeleton rearrangements, which is important for cellular morphogenesis. In addition, AM appears to contribute to neural stem cell growth regulation by allowing cells to pass through mitosis. Consequently, AM may contribute to program stem cells for future clinical uses.

Finally, R. Ramos-Zuniga et al. discussed the ethical implications on the use of stem cells. They explain that every clinical project should take that into account, along with potential clinical applications, the principle of “*primum non nocere*” (first, do no harm). The authors also indicate the importance of keeping a close clinical surveillance to establish the possible risks in the use of stem-cell-based therapies.

With this compendium of cutting-edge review articles and original articles written for experts in the stem-cell field, we hope these in the field of adult neural stem cells will help be helpful and educational for readers.

*Oscar Gonzalez-Perez* *Oscar Gonzalez-Perez* *Jose M. Garcia-Verdugo* *Jose M. Garcia-Verdugo* *Alfredo Quinones-Hinojosa* *Alfredo Quinones-Hinojosa* *Sonia Luquin* *Sonia Luquin* *Graciela Gudino-Cabrera* *Graciela Gudino-Cabrera* *Rocio E. Gonzalez-Castaneda* *Rocio E. Gonzalez-Castaneda*

